# Development of a Novel HS-GC/MS Method Using the Total Ion Spectra Combined with Machine Learning for the Intelligent and Automatic Evaluation of Food-Grade Paraffin Wax Odor Level

**DOI:** 10.3390/foods13091352

**Published:** 2024-04-27

**Authors:** Marta Barea-Sepúlveda, José Luis P. Calle, Marta Ferreiro-González, Miguel Palma

**Affiliations:** Department of Analytical Chemistry, Faculty of Sciences, University of Cadiz, Agri-Food Campus of International Excellence (ceiA3), IVAGRO, 11510 Puerto Real, Spain; marta.barea@uca.es (M.B.-S.); joseluis.perezcalle@uca.es (J.L.P.C.); miguel.palma@uca.es (M.P.)

**Keywords:** food-grade paraffin waxes, food packaging, odor intensity, headspace, gas chromatography–mass spectrometry, total ion spectra, machine learning, Box–Behnken design

## Abstract

The intensity of the odor in food-grade paraffin waxes is a pivotal quality characteristic, with odor panel ratings currently serving as the primary criterion for its assessment. This study presents an innovative method for assessing odor intensity in food-grade paraffin waxes, employing headspace gas chromatography with mass spectrometry (HS/GC-MS) and integrating total ion spectra with advanced machine learning (ML) algorithms for enhanced detection and quantification. Optimization was conducted using Box–Behnken design and response surface methodology, ensuring precision with coefficients of variance below 9%. Analytical techniques, including hierarchical cluster analysis (HCA) and principal component analysis (PCA), efficiently categorized samples by odor intensity. The Gaussian support vector machine (SVM), random forest, partial least squares regression, and support vector regression (SVR) algorithms were evaluated for their efficacy in odor grade classification and quantification. Gaussian SVM emerged as superior in classification tasks, achieving 100% accuracy, while Gaussian SVR excelled in quantifying odor levels, with a coefficient of determination (R^2^) of 0.9667 and a root mean square error (RMSE) of 6.789. This approach offers a fast, reliable, robust, objective, and reproducible alternative to the current ASTM sensory panel assessments, leveraging the analytical capabilities of HS-GC/MS and the predictive power of ML for quality control in the petrochemical sector’s food-grade paraffin waxes.

## 1. Introduction

Food-grade paraffin wax is a petroleum-derived product (PDP) used in a variety of food products as both a preservation material and an additive. For example, it is employed in the production of chewing gum and as a food preservative in cheeses, fruits, and cakes [1,2,3,4,5]. Accordingly, to comply with the U.S. Food and Drug Administration (FDA) standards under 21 CFR 172.886 for its use in food [6] and, 21 CFR 178.3710 for direct food contact [7], paraffin wax for food applications is subjected to a high temperature and pressure hydrogenation process that allows for the removal of both sulfur compounds and aromatic compounds [1,8,9]. Therefore, to ensure food safety, companies responsible for manufacturing and distributing food packaging and additives products must implement quality control programs to ensure product safety and sanitary compliance.

In this context, the petrochemical industry had to adapt both good manufacturing practice principles and legal requirements to implement food safety policies at paraffin wax production facilities. To do this, it was necessary to implement a quality management system, which was based on the American Society for Testing and Materials’ (ASTM International) standardized methods [10]. Among food quality and food-package control parameters, odor intensity is considered one of the most important factors to be evaluated. In this sense, the lack of an undesirable odor is an indicator of final product quality [11]. Therefore, its assessment is very general. For food-grade paraffin wax, the most common method for determining the odor level is governed by the ASTM D1833—Standard Test Method for Odor of Petroleum Waxes [12]—which consists of assessing the intensity of the current odor in paraffin wax through a tasting panel of five experts. Then, the paraffin wax odor is graded on a 5-point scale from 0 to 4, with 0 being odorless (“None”) and 4 being the maximum odor level (“Very strong”). Disadvantages that this method can pose include the need for human resources, problems during the comparisons of the panel testing results among different laboratories or companies, lack of rapid response from sensory tests, and possible health risks to the evaluator due to long-term exposure to undesirable volatile organic compounds (VOCs) and semi-volatile organic compounds (SVOCs) [13]. In this sense, the proper automated and instrumental-based characterization of the global aromatic profile responsible for the organoleptic properties of paraffin wax can facilitate production operations and redirect them toward more effective processes.

Today, there are many analytical techniques used for odor quality control in the food industry and other fields. Among those employed for the characterization of VOCs and SVOCs, methods based on gas chromatography (GC), often combined with mass spectrometry (MS), have been of excellent use due to their high sensitivity and reproducibility [14]. Regarding sample preparation, many options for extracting VOCs and SVOCs can be coupled with the GC/MS system. Some of these options include thermal desorption (TD), solid-phase microextraction (SPME), and headspace generation (HS) techniques, with the last two being the most used [15,16]. In general, the information obtained by GC/MS, the total ion chromatogram (TIC), has been employed to find target compounds that allow for sample characterization [17]. However, this task is time-consuming and requires skilled operators. While this task demands considerable time and expertise, advancements in the GC–MS field have enabled its utilization as a screening or direct method through total ion spectra (TIS). The TIS method involves summing the intensities for each mass-to-charge (*m*/*z*) ratio across the full chromatographic range to generate a time-averaged spectrum [18,19]. This approach serves as an alternative that addresses the challenges linked with retention times and the application of GC/MS as a chemical sensor, functioning similarly to an MS-based electronic nose [20,21,22]. Focusing on this last point, when used as a chemical sensor, the GC/MS operates so that each fragment ion (represented by the *m*/*z* ratio) functions as an individual sensor. The intensity of each ion corresponds to the sensor’s signal. This approach enables the determination of the complete aromatic profile of each sample, which is as distinctive as a fingerprint. Additionally, as TIS is time-independent, it is more suitable for inter-laboratory comparison [22]. The development of GC/MS methods involving the use of TIS results in the acquisition of a large amount of information, and it has become imperative to use advanced machine learning (ML) techniques to transform and manage these data into useful information [22,23,24,25,26]. Unsupervised and supervised machine learning (ML) techniques frequently utilize data from global profiling or screening methods, such as GC/MS with total ion spectra (TIS) and MS-based electronic noses. For instance, unsupervised methods like cluster analysis (CA) and principal component analysis (PCA) are commonly used for pattern recognition [19,24,26,27]. Conversely, supervised techniques such as linear discriminant analysis (LDA), quadratic discriminant analysis (QDA), k-nearest neighbors (kNNs), support vector machine (SVM), random forest (RF), and partial least squares regression (PLSR) are employed for tasks including discrimination, classification, and regression [24,27,28,29,30,31,32]. Moreover, the creation of supervised regression and classification models, which are predictive in nature, can streamline data processing for future samples, thereby simplifying these tasks in the petrochemical and agri-food sectors.

Regarding the application of analytical techniques to the characterization of paraffin wax, Durret (1966) applied GC together with a flame ionization detector (FID), as well as MS, to identify target compounds related to the paraffin smell, indicating that toluene is mainly responsible for the undesirable odor [33]. In turn, Yuan et al. (2013) investigated the presence of toluene and aromatic compounds in the volatile composition of paraffin wax by applying HS-SPME-GC/MS [34]. The results of the study indicated that toluene was the component with the greatest influence on the odor, followed by other minor components such as xylene and other benzene derivatives. Palu et al. (2014) applied GC/MS to characterize de-oiled industrial paraffin wax [35]. On the other hand, in a recent study conducted by our research group [36], we employed TD-GC/MS to identify and quantify VOCs in paraffin waxes used in food processing. In this particular case, the focus was on identifying the VOCs involved in the aroma of the paraffins, and thus, the TIS strategy was not used. On the other hand, Wang et al. (2015) [37] pioneered the use of a gas-sensor electronic nose for discriminating paraffin waxes based on their volatile profiles. They combined this technique with principal component analysis (PCA) for dimensionality reduction and data visualization. Additionally, kNNs, SVMs, and multilayer perceptrons were employed to classify the different qualities of petroleum waxes. For their part, Men et al. (2018) [13] extended the use of an electronic nose integrating a gas sensor network with ML techniques such as SVM, RF, and extreme learning machine (ELM) to enhance odor analysis systems for paraffin wax, yielding commendable outcomes. Their work introduced an innovative screening method for the automatic assessment of odor levels in paraffin products. Despite these excellent results, the reliance on gas sensor-based systems introduces inherent limitations, including issues with sensor stability and sensitivity. To overcome these challenges, there is an increasing interest in investigating alternative technologies, such as HS–MS electronic noses or HS-GC/MS using the TIS strategy, that offer greater sensitivity and robustness. These methods provide a more detailed and comprehensive aroma profile of samples, making them particularly well-suited for critical quality assessments, such as odor evaluation in paraffin waxes.

Therefore, after all the above, in this study the integration of TIS with HS-GC/MS was explored to assess its efficacy as an electronic nose for capturing the global aroma profile of food-grade paraffin waxes. This innovative approach aims to capture the comprehensive aroma profile of food-grade paraffin waxes, addressing the limitations of traditional gas-sensor electronic noses and the targeted analyte approach typically employed in GC/MS with TIC. This approach has been successfully applied previously to other petroleum-derived products [22]. By leveraging TIS, this research seeks to enhance the objectivity and reproducibility of sensory analysis methodologies, offering a viable alternative to the current ASTM standards used in the petrochemical industry. For this purpose, a Box–Behnken design (BBD) using response surface methodology (RSM) was applied to variables related to headspace generation. In the present investigation, GC/MS is used as a multi-sensor device with all the advantages that this brings. Therefore, TIS was proposed as a new robust approach to determine the optimal conditions required to best distinguish and quantify odor levels in paraffin waxes. Furthermore, the final goal of this study is to apply the developed method to paraffin wax samples with different odor levels, to detect and distinguish the degree of odor intelligently and simply. To do so, TIS was combined with several ML tools to establish the optimal algorithms to obtain better efficiency from the results and information contained in the data. The best-performing models generated were employed to be integrated into an interactive web application for sharing purposes.

## 2. Materials and Methods

### 2.1. Paraffin Wax Samples

In this study, paraffin wax samples provided by Compañia Española de Petroleos, S.A.U., (CEPSA) San Roque refinery (Cadiz, Spain) were used. Specifically, two types of paraffin waxes obtained from crude oil were studied: (a) 5 independent lots taken in different years of fully refined (hydrogenated) with a food-grade certification, and (b) 5 independent lots taken in different years of non-hydrogenated, unfit for use in agri-food products. The selection of these two types of paraffins was based on two key factors: (1) during the training of expert sensory panels, practice mixtures are prepared using both hydrogenated and non-hydrogenated paraffins to familiarize the panel with the characteristics of each type, and (2) paraffins that have undergone hydro-treatment processes, which were not completed successfully, will exhibit an odor.

#### Odor Levels Preparation

Food-grade and non-hydrogenated paraffin wax samples were mixed and heated at 100 °C in odorless sealed bottles at different percentages to obtain a range of samples with different odor levels. Specifically, mixtures of food-grade paraffin wax with added non-hydrogenated paraffin wax were prepared in ratios of 25%, 50%, and 75%. In addition, food-grade paraffin (0%) and non-hydrogenated paraffin (100%) were also analyzed. In this way, five different odor levels linked to the five base levels of the ASTM D1833 [12] standard were established. Therefore, 0% corresponds to a grade 0 odor (“*None*”), 25% to a grade 1 (“Slight”), 50% to a grade 2 (“Moderate”), 75% to a grade 3 (“Strong”) and 100% to odor level 4 (“Very Strong”). Each of the mixtures prepared was analyzed in triplicate. In this way, a total number of samples equal to *n* = 75 (i.e., 5 lots × 5 percentages × 3 replicates) was obtained. Before the analyses, the samples were sealed with aluminum foil and subsequently stored in single odorless plastic containers according to their odor grade to avoid cross-contamination. 

To be analyzed, the paraffin waxes were labeled as follows: first, the percentage of non-hydrogenated paraffin wax content over food-grade paraffin; this was followed by the sample lot, i.e., A, B, C, D, or E; and finally, the replicate analysis of that sample, i.e., R1, R2, or R3 was labeled. For example, the first replicate analysis for the sample from lot A with 25% non-hydrogenated paraffin wax content would be labeled as 25%_A_R1 and the second replicate analysis for the sample from lot B with 50% non-hydrogenated paraffin wax content would be labeled as 50%_B_R2.

### 2.2. HS-GC/MS Acquisition

HS-GC/MS analyses were performed using an HS unit (AOC-6000; Shimadzu Scientific Instruments, Kyoto, Japan) coupled with a GC/MS with a triple quadrupole (Q3) (GC/MS TQ8040; Shimadzu Scientific Instruments, Kyoto, Japan), which comes integrated with the workstation software, GCMSsolution Version 4.52, for Shimadzu’s GCMS-TQ series gas chromatography-mass spectrometers. No pretreatment was applied to the samples. The optimized conditions for the generation of the headspace were as follows: 10 mL HS vials (Agilent CrossLab, Santa Clara, CA, USA) containing the paraffin waxes (0.5825 g) were placed directly into the oven of the autosampler for both heating (140 °C) and shaking (250 rpm (5 s on and 2 s off)) for 5 min to generate the headspace (HS). The syringe was kept 5 °C above the incubation temperature to avoid condensation. The HS conditions presented here were optimized using a BBD with RSM. See Section 2.4. for selection and description. The GC was equipped with a BPX5 capillary column (length 30 m; internal diameter 0.25 mm; film thickness 0.25 μm; SGETM Analytical Science, Melrose Park, North West Shelf, Australia). Helium (purity grade 5N) was the carrier gas with a column flow rate of 0.94 mL/min and with an average linear velocity of 35.0 cm/s. The injection was split in a 15:1 ratio. A 500 µL of the generated HS was injected into the injection port (150 °C). The GC oven temperature program started at 40 °C (held for 5 min). Subsequently, the temperature was increased by 3 °C per minute to 50 °C, followed by an increment to 270 °C at 40 °C/min (held for 2 min). The mass spectrometer ion source and interface temperatures were 200 °C and 275 °C, respectively. In turn, the ionization mode used was electron impact at 70 eV. Mass spectra were scanned between 50 and 600 mass-to-charge ratios (*m*/*z*). The total time of the GC/MS program employed was 15.83 min.

### 2.3. Optimization of Conditions

The different variables affecting the signal of the TIS are mainly related to the specific conditions used to generate the HS of the VOCs and SVOCs. Based on the literature, the factors that influence HS generation most are incubation temperature, incubation time, agitation, and sample quantity [38]. In the present study, a BBD–RSM was applied to optimize the incubation temperature, agitation, and sample quantity. On the other hand, the incubation time was evaluated subsequently using a kinetic study based on a univariant design.

#### 2.3.1. Experimental Design

In the present work, this BBD with RSM methodology was used to obtain the most suitable working conditions for the generation of HS in the paraffin wax samples. BBD is a type of factorial design that is highly efficient for exploring quadratic response surfaces and constructing second-order polynomial models without involving all the combinations of the levels of each factor. This method strategically selects a set of points in the design space to efficiently estimate the coefficients of the model, thus assessing the interaction and quadratic effects of the variables involved. BBD is particularly advantageous when the number of experiments needs to be minimized due to time or cost constraints. On the other hand, RSM is a collection of mathematical and statistical techniques that are useful for modeling and analyzing problems where several variables influence a response. RSM is used to find an optimal response by fitting a regression model to the data obtained from carefully designed experiments. This methodology aids in understanding the effects and possible interactions among the design factors.

During the BBD-based optimization process, two types of variables were considered: the factors or independent variables and the response variable or dependent variable. Regarding the independent variables, three factors were selected to carry out the optimization: incubation temperature (°C), agitation (rpm), and sample quantity (g). The incubation time was maintained at 10 min during all the experiments. Considering the difference in unit, level, and range between each factor, the levels were normalized from −1 to +1 to obtain a uniform response. Thus, every factor has three levels: a lower level (−1), an intermediate level (0), and an upper level (1). The independent variables and their levels are presented in Table 1. The ranges evaluated for each factor were established based on the objective of this study. In the case of incubation temperature, a minimum value of 100 °C was used to ensure the complete melting of the sample and to extract both VOCs and SVOCs from it. The range studied for the incubation temperature was from 100 to 140 °C. The speed to be evaluated in the sample agitation was studied between 250 and 750 rpm. On the other hand, small sample quantities to be studied were selected based on the research group’s previous experience in using the technique with other PDPs and to avoid detector saturation. The range used for the quantity of sample was from 0.2 to 0.6 g.

The complete design consisted of 18 experiments with 6 replicates at the center point. All trials were conducted in random order. The determination of the optimal HS conditions was performed by RSM. In this case, the target response to be maximized was the differences between the odor grades of the paraffin wax samples. For this purpose, two types of samples were used for the 18 BBD experiments: paraffin wax with a “Slight” odor grade (25% non-hydrogenated paraffin wax content) and paraffin wax with a “Very Strong” odor grade (100% non-hydrogenated paraffin wax content).

The TIS normalized to the maximum signal (base peak) was obtained for each sample and analyzed under the specific BBD conditions. Using this information, the Euclidean distance between the paraffin wax sample with 25% non-hydrogenated content and the paraffin wax sample with 100% non-hydrogenated content was calculated for each of the experiments performed. The response variable was, therefore, the Euclidean distance between the two samples. This dependent variable was used to develop a mathematical model with a second-order polynomial function (Equation (1)):

(1) $$Y=\beta_{0}+\sum_{i=1}^{k}\beta_{i}x_{i}+\sum_{i=1}^{k}\beta_{ii}x_{ii}^{2}+\sum_{i<1}^{k}\beta_{ij}x_{i}x_{j}+\epsilon$$

In this equation, *Y* represents the response; *β*_0_ is the model constant; *x* represents each of the factors considered; *β_i_* is the coefficient of each main effect; *β_ii_* is the coefficient of the quadratic factors representing the curvature of the surface; *β_ij_* is the coefficient corresponding to the interactions between *I* and *j*; and, finally, *ϵ* represents the residual value due to random error. The suitability of the model obtained was evaluated from the resulting lack-of-fit value and the determination coefficient (R^2^), and its statistical significance was measured using an analysis of variance (ANOVA).

#### 2.3.2. Kinetic Study

The migration of VOCs and SVOCs in the HS takes a finite amount of time, which is determined by the time required for molecular diffusion in the sample phase and the transfer of the compounds to the gas phase. 

Thus, to achieve the complete extraction of VOCs and SVOCs, thermodynamic equilibrium must be reached. Because the kinetic behavior of this process is difficult to mathematically model, it is necessary to conduct a study to evaluate the kinetics of the equilibrium time. The kinetics of incubation time was evaluated using univariate design methodology, setting the optimal conditions obtained for sample quantity, incubation temperature, and agitation, and varying the incubation time. A total of five incubation times were tested: 5 min, 10 min, 15 min, 20 min, and 25 min. Times less than 5 min were not studied to ensure complete sample fusion and the creation of a repeatable and reproducible HS. Furthermore, times longer than 25 min were not investigated because the developed method must be fast enough to be used in routine analysis. The kinetic study experiments were carried out with a 25% non-hydrogenated content paraffin wax sample and a 100% non-hydrogenated content paraffin wax sample. The same response variable used in the rest of the HS working conditions optimization employing BBD–RSM was selected as the response variable to be evaluated.

### 2.4. Data Analysis

#### 2.4.1. Total Ion Spectra

The HS-GC/MS data were used following the procedure described by Sigman et al. (2008) to obtain the TIS [18]. This procedure implies that the second-order chromatographic data are collapsed along the retention time dimension, resulting in a profile of relative intensities of ions in the total sample. Each of the obtained TIS was normalized using the base peak normalization method, also known as normalization to the maximum signal. This normalization involves dividing the intensity of each *m*/*z* ratio by the intensity of the base peak of that sample. Finally, the normalized TIS data matrix was oriented so that each row consisted of the intensity of a single sample in all variables, yielding a matrix D_mxn_, where *m* is the number of *m*/*z* values and n is the number of samples. In this case, the total number of *m*/*z* was 501 (*m*/*z* 50–550) and the number of samples equaled 75.

#### 2.4.2. Machine Learning Algorithms

Two unsupervised ML algorithms, namely hierarchical cluster analysis (HCA) and PCA, were chosen for the current study to perform an exploratory study for finding patterns and grouping trends in the normalized dataset (D_501×75_), specifically to determine if samples tend to cluster based on their odor levels. Furthermore, the use of various supervised ML algorithms was reviewed and evaluated for the creation of predictive models. Specifically, the Gaussian SVM and RF algorithms were investigated for the construction of classification models that allow differentiating paraffin waxes according to odor intensity. On the other hand, the PLSR, RF regression, and Gaussian SVR algorithms were investigated for the construction of regression models that allow the assessment of the odor level percentage in the paraffin wax samples. The classification models’ performance was assessed using the metrics of accuracy and kappa. For its part, the coefficient of determination (R^2^) and the root mean square error (RMSE) were the metrics evaluated in the regression models. The normalized dataset (D_501×75_) was randomly split (split = 0.7) into a training set and a test set for the development of the quantification and regression models. Each algorithm’s training and hyperparameter optimization were performed using the training set. Then, the created models were validated against the test set for their part. To reduce model overfitting, all models were constructed using 5-fold cross-validation (CV) during hyperparameter tuning and training. For the classification models, a total of five a priori groups were established based on the odor ASTM D1388 category (“None”, “Slight”, “Moderate”, “Strong”, and “Very Strong”), whereas for the regression, the prior groups (“0%”, “25%”, “50%”, “75%”, and “100%”) were associated with the percentage of non-hydrogenated paraffin wax over food-grade paraffin wax.

### 2.5. Software

GCMSsolution (GCMSsolution Version 4.52, Shimadzu Scientific Instruments, Kyoto, Japan) workstation software was used to perform the HS-GC/MS analyses and extract the data in a .cfd extension file. The raw data (3D data matrix in .TIC file extension) were obtained employing the open-source software AMDIS (automated mass spectral deconvolution and identification system; version 2.73; 25 April 2017) from the National Institute of Standards and Technology (NIST). Such raw data were employed to create the TIS for each sample. The 2D data matrix constructed with the TIS of the samples was saved in .xlsx format. Statgraphics Centurion XVI.I (Statgraphics Technologies Inc., The Plains, VA, USA; version 16.1.03) was used for the RSM-BBD development and the analysis of optimal HS conditions. The data visualization and application of ML techniques were carried out using the open-source programming language R (version 4.1.2, Boston, MA, USA) [39]. HCA was performed using the *hclust* function of the stats package (version 4.1.2) [39]. Linkage method selection for the HCA was established by computing the agglomerative coefficient of different linkage methods (Average, Single, Complete, and Ward) using the *agnes* function of the cluster package (version 2.1.3) [40]. The HCA results were represented in a dendrogram using the *fviz_dend* function of the *factoextra* package (version 1.0.7) [41]. The ML models were developed using the *trainControl* and *train* functions of the *caret* package (version 6.0-93) [42]. The metrics of the generated regression models were established with the built-in functions in the *MLmetrics* package (version 1.1.1) [43]. The most important variables in the generation of the PLS and RF models were extracted with the *varImp* function of the *caret* package (version 6.0-93) [42]. All the other graphs generated for data visualization were built using the *ggplot2* (version 3.3.6) [44] and the *graphics* (version 4.1.2) [39] packages. The interactive web application was developed using the *shiny* package (version 1.7.2) [45].

## 3. Results and Discussions

### 3.1. Method Optimization

#### 3.1.1. Box–Behnken Design with RSM

The HS factors that can significantly impact the efficiency with which VOCs and SVOCs are transferred from a sample to the gas phase include a trio of critical variables: the incubation temperature, agitation speed, and the quantity of the sample used. These variables were meticulously explored across a range of settings: incubation temperature (X_1_: 100, 120, and 140 °C), agitation (X_2_: 250, 500, and 750 rpm), and sample amount (X_3_: 0.2, 0.4, and 0.6 g). To ensure a standardized comparison, all analyses were conducted with the incubation time uniformly set at 10 min. This rigorous examination facilitated the evaluation of these variables’ influence on transfer efficiency to systematically study the interactions between them. The BBD and the response, which is expressed as the Euclidean distance between the normalized TIS of the paraffin wax with 25% content in non-hydrogenated and the paraffin wax with 100% content in non-hydrogenated, are shown in Table 2. 

A comprehensive ANOVA was performed to assess the effects of the factors and their potential interactions on the experimental outcomes. The detailed results of the ANOVA are shown in Table 3. This table presents a thorough breakdown of the coefficients for each parameter within the second-order polynomial equation derived from the study, alongside their statistical significance, quantified through *p*-value. Based on this significance, it can be determined which factors and/or interactions have a more significant influence on the response. Therefore, only factors and/or interactions with *p*-values less than 0.05 were considered relevant to the response at the established significance level (95%). As can be seen in Table 3, only the linear term for incubation temperature and sample quantity was significant (*p*-value < 0.05). Incubation temperature is an important factor as it must be sufficient to facilitate molecular diffusion and migration of VOCs and SVOCs into the gas phase. In this case, incubation temperature positively affected the response (b_1_ = 0.0245). This implies that the two groups were discriminated against more effectively as the incubation temperature increased. The sample quantity is another important factor in creating an optimal HS, as it is directly related to the analyte concentration entering the gas phase. The results obtained show that the sample quantity has a positive effect on the response (b_3_ = 0.0109). This means that discrimination between the two groups was more successful when the sample quantity was at the upper end of the study range. 

**Figure 1:**
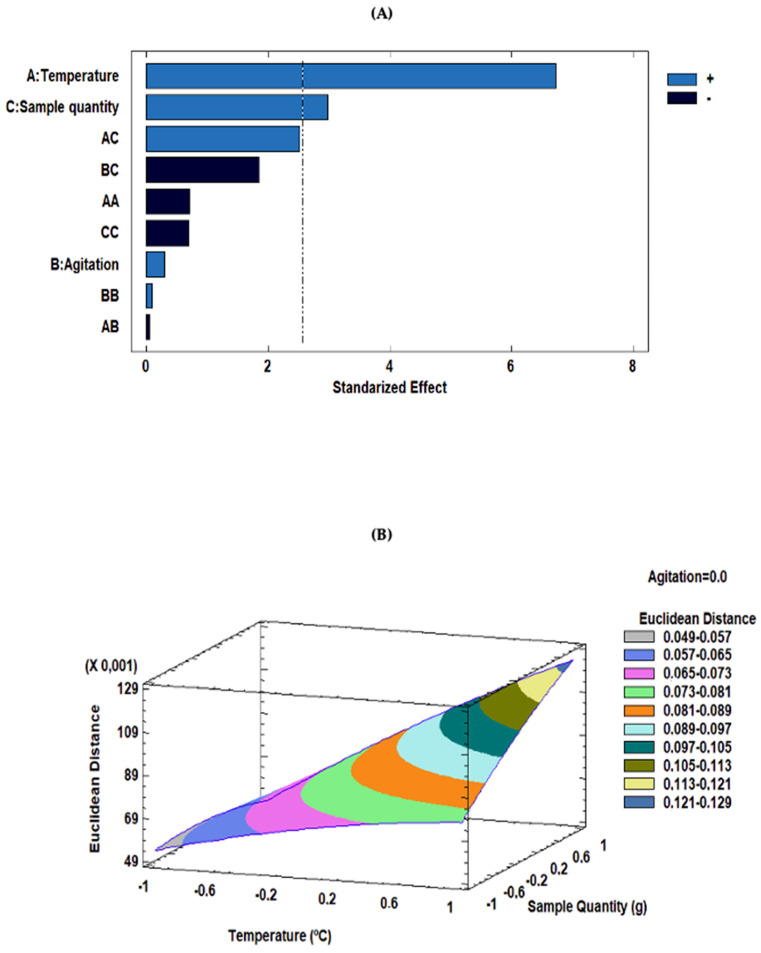
(**A**) Pareto chart of standardized effects; (**B**) 3D surface plot of the Box–Behnken design to represent the influence of sample quantity and temperature on the response variable.

The results obtained show that the sample quantity has a positive effect on the response (b_3_ = 0.0109). This means that discrimination between the two groups was more successful when the sample quantity was at the upper end of the study range. For a visual representation of the effects and their combinations, the standardized Pareto chart is displayed in Figure 1. The effect of each factor or interaction of factors is represented graphically by bars arranged in decreasing order of effect on the response. Based on the coefficients of the factors and interaction effects (Table 3), a quadratic polynomial regression model can be obtained to predict the response variable as a function of the independent variables (Equation (2)). The full equation can be reduced by considering only the significant factors and interactions (*p*-value < 0.05). The reduced equation is represented in Equation (3):

(2) $$\begin{array}{c} Y=0.0831+0.0245\cdot X_{1}+0.00111\cdot X_{2}+0.0109\cdot X_{3}-0.00350\cdot {X_{1}}^{2}-0.000304\cdot X_{1}X_{2}\;+\\ 0.0130\cdot X_{1}X_{3}+0.000522\cdot {X_{2}}^{2}-0.00958\cdot X_{2}X_{3}-0.00346\cdot {X_{3}}^{2} \end{array}$$

*Y* = 0.0831 + 0.0245·*X*_1_ + 0.0109·*X*_3_ (3)

where *Y* is the Euclidean distance between the normalized TIS of the paraffin wax with 25% content in non-hydrogenated and the paraffin wax with 100% content in non-hydrogenated and *X*_i_ (*X*_1_, incubation temperature; *X*_2_, agitation; *X*_3_, sample quantity).

A lack-of-fit test was carried out to assess whether the selected model was adequate to describe the observed data or whether a more complex model was required. The results showed that the *p*-value of the lack-of-fit (0.436) was greater than 0.05. Therefore, the model was considered satisfactory for explaining the observed data at the 95% confidence level. The comparison between experimental and predicted values, as shown in Figure S1 (Supplementary Materials), indicated a good agreement demonstrated by an R^2^ of 88.75%. Therefore, the model was suitable for use in estimating the response for optimization. According to the fitted model and using the RSM, a 3D surface plot was generated to predict the relationship between the independent and dependent variables. Concretely, Figure 1B illustrates the combined effects of incubation temperature and sample quantity on the response variable. As can be seen in Figure 1B, maximization of the response variable was found to be obtained at coordinate +1 (140 °C) for incubation temperature, that means the maximum temperature allowed by the instrument. On the other hand, a value of +0.9708 (0.5825 g) for the sample quantity. Therefore, the optimal final conditions were an incubation temperature equal to 140 °C, sample quantity of 0.5825 g, and agitation at 250 rpm.

#### 3.1.2. Kinetic Study of the Optimal Conditions

**Figure 2:**
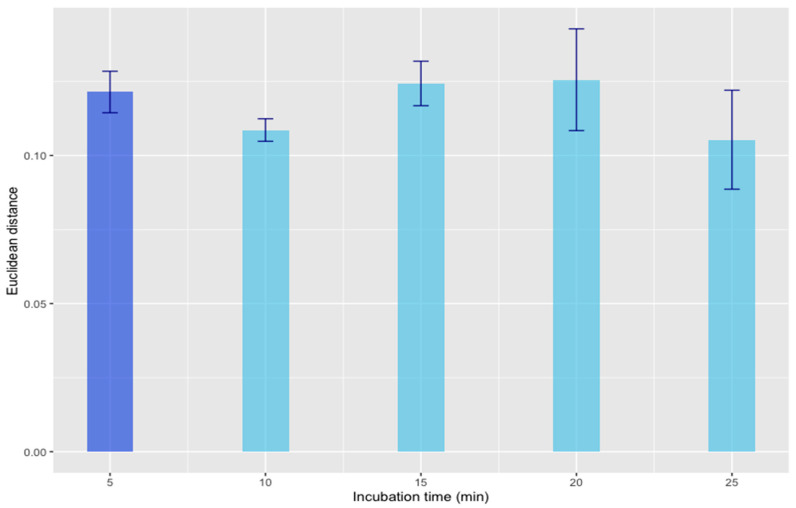
Average Euclidean distances calculated from the normalized TIS between two samples (*n* = 3) and standard deviations using the optimized conditions.

The solid-gas equilibrium was evaluated using the optimal predefined conditions (incubation temperature = 140 °C; sample quantity = 0.5825 g; agitation = 250 rpm) and varying the incubation time from 5 to 25 min (in 5-min increments). Experiments were carried out in triplicate for both the 25% non-hydrogenated paraffin wax and the 100% non-hydrogenated paraffin wax, thus giving a total of 30 analyses. The response variable was the same as the previously evaluated one, i.e., the Euclidean distance calculated from the normalized TIS between the two samples. The graph in Figure 2 shows the evolution of the mean (*n* = 3) of the response variable in each incubation time evaluated with its standard deviation (s.d.). To determine the existence of a statistically significant difference between the response variable values as a function of incubation time, an ANOVA was performed. The results of this analysis indicated that there were no statistically significant differences (*p*-value < 0.05) at a confidence level of 95%. In this way, to minimize the analysis time for obtaining a rapid routine method, it was concluded that the optimal incubation time would be set to 5 min.

### 3.2. Repeatability and Intermediate Precision of the Method

The precision of the developed HS method was evaluated according to repeatability and intermediate precision. Repeatability was calculated as the closeness between the results of experiments performed on the same day under the same conditions. Intermediate precision was calculated as the closeness between results on different days. Specifically, to evaluate the repeatability, a total of 6 analyses were carried out and completed on the same day under optimal conditions for each of the 2 samples (paraffin wax with a content of 25% non-hydrogenated and paraffin wax with a content of 100% in non-hydrogenated). A total of 24 samples were analyzed (12 samples of paraffin wax with 25% content in non-hydrogenated and 12 samples of paraffin wax with 100% content in non-hydrogenated). The Euclidean distance between the normalized TIS of the two samples in the repeatability and intermediate precision experiments was calculated. The coefficient of variation (C.V.) between Euclidean distances was used as the statistical measure to assess their similarity. The C.V. for repeatability was 8.96% and 7.04% for intermediate precision. Since both values were within acceptable limits (10%), the developed method was found to be reproducible and to have an adequate intermediate precision.

### 3.3. Machine Learning Evaluation

Once the HS method was optimized, it was applied to discriminate and quantify the degree of odor in paraffin wax samples. To extract the maximum information from the normalized TIS, the first step was to study the clustering tendencies of the samples to evaluate whether they occurred according to the odor level. Then, different supervised ML algorithms were evaluated using the full dataset to obtain accurate and optimized predictive models to discriminate (classifiers) and quantify (regression models) the odor level of the samples.

#### 3.3.1. Exploratory Study

**Figure 3:**
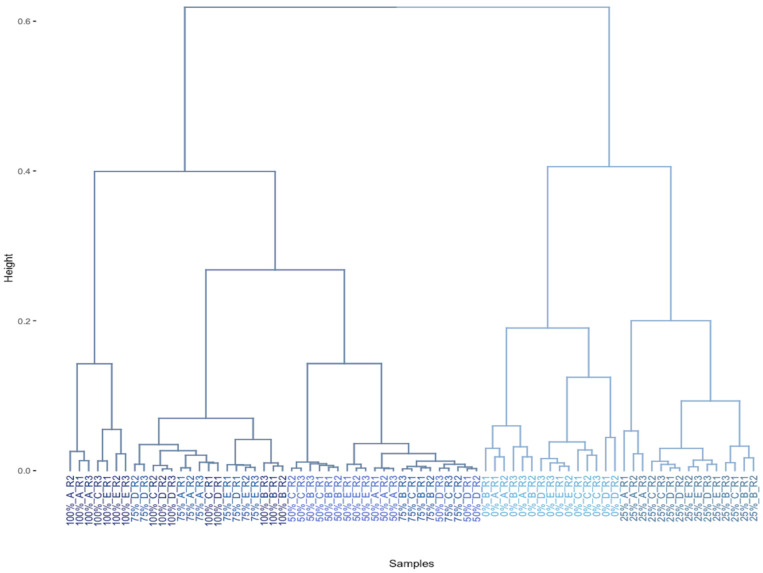
Dendrogram obtained from the HCA using the Euclidean distance and Ward’s method. The paraffin wax samples (*n* = 75) were colored in different blue tones according to their percentage of odor.

The initial exploration of paraffin wax samples’ tendency to cluster based on their odor grade involved employing HCA. Ward’s method was specifically chosen as the linkage method, complemented by the utilization of Euclidean distance as the distance metric. The selection of linkage method was chosen through a comparison of agglomerative coefficients derived from various methods, including Average, Complete, Simple, and Ward. A higher agglomerative coefficient, nearing 1, suggests a more robust clustering structure. Remarkably, among the methods evaluated, Ward’s method exhibited the highest agglomerative coefficient at 0.98, indicating its superior ability to delineate distinct clusters effectively. The outcomes of the HCA analysis were vividly presented through the dendrogram illustrated in Figure 3.

**Figure 4:**
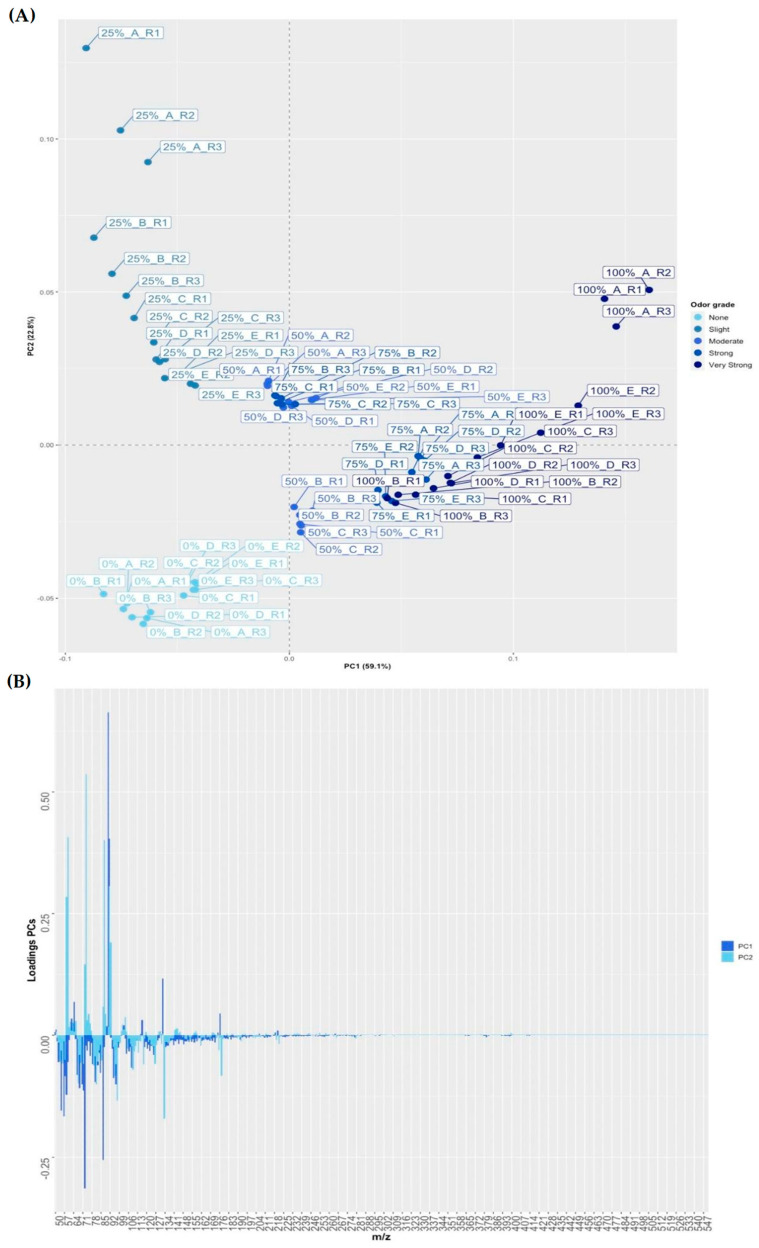
(**A**) Score plot PC1 vs. PC2 for all paraffin wax samples (*n* = 75); (**B**) Loadings obtained for each *m*/*z* in the first two PCs in the PCA.

A PCA was conducted to gain further insights into the spectral ranges influencing the observed results. Figure 4A displays a scores plot for the first two principal components (PC1 and PC2) across all samples (*n* = 75), while Figure 4B illustrates the loadings for PC1 and PC2. Together, PC1 and PC2 accounted for 59.1% and 22.7% of the data variance, respectively, totaling a cumulative variance of 81.8%. These components were instrumental in differentiating the wax samples based on their odor intensity levels. Specifically, Figure 4A shows PC1 differentiating samples with 0% and 25% non-hydrogenated paraffin wax (negative loadings on PC1) from those with 50%, 75%, and 100% wax content (positive loadings on PC1). Conversely, PC2 distinctly separated the 0% and 25% odor grade samples, with the former showing negative loadings and the latter positive. A less distinct trend was observed among the 50%, 75%, and 100% non-hydrogenated content samples. These PCA results align with those from the hierarchical cluster analysis. The loadings plot in Figure 4B indicates that the *m*/*z* range, particularly *m*/*z* 91, significantly contributed to the separation on PC1, exhibiting a notable weight value of 0.66. This *m*/*z* is usually related to aromatic alkylbenzenes. This would agree with previous studies by Durret (1966) and Yun et al. (2013) [38,39], who identified that the odor in paraffin waxes derived mostly from aromatic alkylbenzenes such as toluene. However, it should be noted that in TIS the *m*/*z* contributions came from the total sum of intensities for each *m*/*z*. For this reason, the origin of the responsible compounds cannot be precisely defined. On the other hand, it was observed that the *m*/*z* with the highest weight in PC2, at 0.54, is *m*/*z* 73. The information obtained on the spectral signals can help to understand which ones are responsible for grouping the samples according to their odor level.

#### 3.3.2. Classifiers

##### Gaussian SVM Classifier

First, the hyperparameters C and σ were optimized using the training set to create an SVM classifier with a Gaussian kernel implementation. Here, we performed the optimization using the grid search method with the exponential growth of both C and σ. In this case, log_2_C and log_2_σ ranged from −10 to 10 in 0.5 intervals. Each combination of parameter selections was evaluated using a 5-fold CV, and the smallest parameter value with the highest 5-fold CV accuracy was selected as optimal. Figure S2 (Supplementary Materials) shows the contour plots where the optimization of C and σ using the grid search method is displayed. It can be observed that the value of the 5-fold CV accuracy increased as log_2_C, and, thus, C, increased (Figure S2). The C parameter controls the number and severity of margin (and hyperplane) violations allowed in the fitting process. Higher values of C have smaller margins, so fewer observations are support vectors (SVs), and the resulting classifier has less bias, but more variance. In this work, the optimal C value was set to 128 (log_2_C = 7). Otherwise, the 5-fold CV accuracy grew as the log_2_σ value increased and, consequently, as σ increased. The value of σ controls the kernel’s behavior and increasing its value correspondingly increases the model’s flexibility. In this case, the optimal value of σ was set to 724 (log_2_σ = 9.5). Additionally, the number of SVs used for the optimized model was set to 42. After hyperparameter tuning, the model was trained with the optimal values of C and σ obtained using the training set and applying a 5-fold CV, yielding 94.4% 5-fold CV accuracy and 0.93 5-fold CV kappa. The performance of the Gaussian kernel SVM classifier generated was evaluated and validated using the test set, showing an accuracy of 100% and a kappa of 1 which confirmed the excellent performance of the generated Gaussian SVM classifier in discriminating the paraffin waxes according to their odor levels.

##### RF Classifier

The optimal hyperparameter values of mtry and ntree were first established using the training set (D_501×55_) for building the RF model. The square root of the total number of predictors was used as the optimal value of mtry and equals 22.38 (501 predictors). For its part, the number of decision trees is not a critical hyperparameter, because adding a large number of decision trees is not associated with the risk of overfitting. However, the analyst must determine its value in advance to stabilize the error and minimize the loss of computational resources. To determine the number of decision trees to use, the values of ntree in this study were set from 2 to 100 with 2 tree intervals, and the highest stable accuracy of 5-fold CV was taken as the endpoint. 

The results are shown graphically in Figure S3 (Supplementary Materials), which demonstrates that the accuracy rate tended to be stable in 16 decision trees and stayed up to 54. From this number of decision trees, a slight increase and decrease in the 5-fold CV accuracy were observed. The accuracy rate then stabilized again from 78 decision trees and was maintained until 100. In this sense, the number of decision trees was set at 100 because it is a large enough number to stabilize the error without a considerable computational cost. Optimal values determined for mtry and the number of decision trees were then used to train the RF model with the training set applying a 5-fold CV. The results from the RF model during training showed a 5-fold CV accuracy of 87.1% and a 5-fold CV kappa of 0.84. Additionally, the OOB estimate of the error rate was 23.64%. The model’s performance was then evaluated and validated using the test set, obtaining an accuracy of 85.0% and a kappa of 0.81. The RF model also presented a lower performance in terms of paraffin wax discrimination as a function of odor intensity compared to that obtained by the Gaussian SVM classifier. Specifically, attending to the confusion matrix obtained for the test set, the model misclassifies, on the one hand, one “Moderate” odor sample into a “Very Strong” odor, and, on the other hand, two “Very Strong” odor samples into “Moderate”.

On multiple occasions, using a large number of predictors is not equivalent to higher model performance, since the model may capture existing noise in the data and redundant information. As such, the selection of a subset of variables to obtain a better fit with the algorithm employed can be presented as a solution. Unlike the SVM algorithm, the RF algorithm allows for the extraction of which predictors (i.e., *m*/*z* intensities) have a higher relative importance in the classification of the samples. For this purpose, the *varImp* function of the caret package was used to estimate the contribution of each variable to the model. In the case of RF, this function calculates the prediction accuracy on the out-of-bag portion of the data for each tree. Subsequently, the same is completed after permuting each predictor variable. Finally, the difference between the two accuracies is averaged over all trees and normalized by the standard error. Specifically, eight *m*/*z* (79, 92, 95, 97, 118, 157, 188, 221) were selected with relative importance higher than 70% (Supplementary Figure S4). A one-way ANOVA was performed for each of the variables selected by the RF model on the 5 types of odor samples, and all of them showed statistically significant differences at a 95% confidence level. Therefore, the fit of the RF algorithm when using the training and test sets reduced to these eight *m*/*z* intensities was studied and evaluated. Here, the value of mtry was set to 2828 and the number of decision trees to 100. The results showed an accuracy of 94.36% and a kappa of 0.93 for the 5-fold CV set, with an OOB error rate of 7.27%. In addition, the model performance was evaluated using the test set reduced to the selected variables, obtaining an accuracy of 95.0% and a kappa of 0.94. The improvement in accuracy and kappa in both the 5-fold CV and test sets and, likewise, in the OOB error rate is noteworthy. In this sense, for the discrimination of paraffin waxes according to their odor grade, it would be necessary to use this RF-reduced model to obtain a precise classification. 

**Figure 5:**
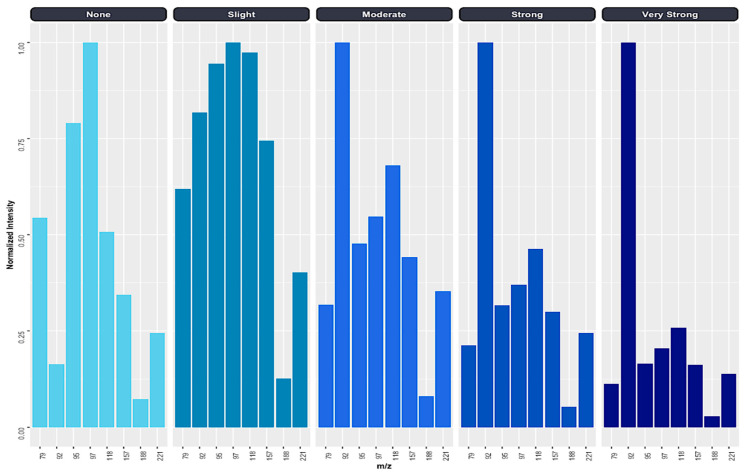
Graphical display of the characteristic spectralprint for each odor intensity in paraffin waxes.

The results obtained through the ANOVA and for the RF-reduced model indicate that the intensities of these eight *m*/*z* could be used to generate a characteristic spectralprint of the different odor degrees of paraffin waxes for the rapid and visual discrimination between them. Figure 5 shows the spectralprints represented in bar charts using the mean intensities values normalized to the maximum of the signal for each of the eight *m*/*z*. According to the results, differences were observed in terms of intensity for these *m*/*z* depending on the degree of odor. Paraffin waxes without odor (“*None*”) and with a “*Slight*” odor presented their maximum intensity at *m*/*z* 97, while paraffin waxes with a “*Moderate*”, “*Strong*” and “*Very Strong*” odor presented their maximum intensity at *m*/*z* 92. Between the paraffin waxes of “*None*” odor and those of “Slight” odor, there were differences in the level of intensity in the remaining 7 *m*/*z* (*m*/*z* 79, 92, 95, 118, 157, 188, and 221), highlighting the *m*/*z* 92, 118, and 157, which presented intensities equal to or higher than 0.75 for the “Slight” odor paraffin waxes and below 0.5 for the “None” odor paraffin waxes. On the other hand, the “Moderate”, “Strong” and “Very Strong” odors differed from each other in a decrease in the intensity of the remaining *m*/*z* (*m*/*z* 79, 95, 97, 118, 157, 188, and 221) as the degree of odor in the paraffin wax increased.

#### 3.3.3. Regression Models

For this section, the results obtained from the developed models have been summarized in Table 4. 

##### PLSR

**Figure 6:**
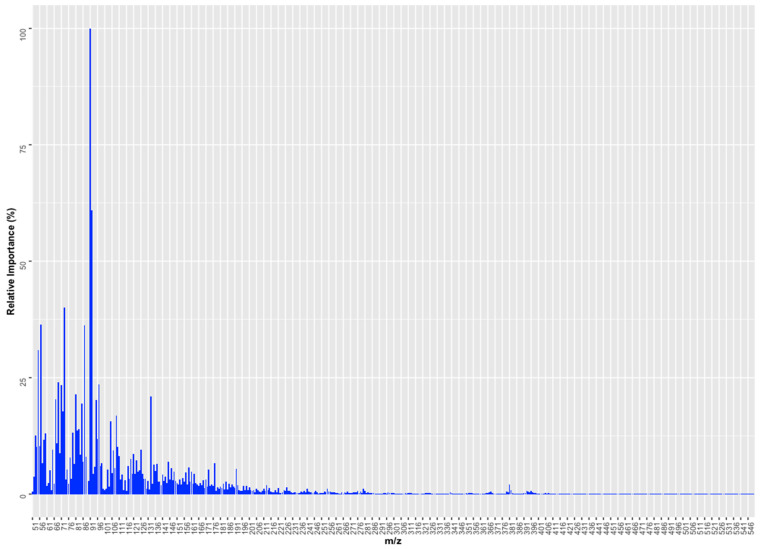
The relative importance of *m*/*z* in the creation of the PLSR model.

The optimal number of components for the PLSR model was determined by a 5-fold CV on the training set. The chart in Figure S5 (Supplementary Materials) illustrates the progression of the RMSE relative to the number of components utilized. The optimal component count for the PLSR model was determined to be 7, based on achieving the lowest RMSE, which was 9.797. This setup also resulted in an R^2^ value of 0.9330 from a 5-fold cross-validation, as detailed in Table 4. These 7 components accounted for 99.40% of the variance in predictors and 93.71% of the variance in response. The performance of the generated model was evaluated using the test set, obtaining RMSE and R^2^ values equal to 9.227 and 0.9328 (Table 4), respectively. Due to the PLSR algorithm’s nature, the variables’ importance in the generated model can be determined. This information can be used to facilitate the understanding of the results. To establish the relative importance of the variables, the varImp function of the caret package was used. For PLSR, the measure of the importance of the variable is based on the weighted sums of the absolute regression coefficients. A graphical representation of the relative importance of the predictors is shown in Figure 6, which shows that *m*/*z* 91 had a relative importance of 100%, followed by *m*/*z* 92 with 61%. These results agreed with those observed in the PCA, where *m*/*z* 91 had a higher weight in PC1 and with those obtained in the RF classifier, where *m*/*z* 92 had a percentage of relative importance above 70%.

##### RF Regression

For building regression models based on the RF algorithm, the value of *mtry* and the number of decision trees need to be set. The parameter mtry in regression problems is usually set to p/3, so in this case, it was established at a value of 167. For their part, to determine the number of decision trees to use, the values of ntree were explored from 2 to 100 with 2 tree intervals using the training set, and the lowest stable RMSE value in the 5-fold CV was set as the endpoint. The results are shown graphically plotted in Figure S6 (Supplementary Materials), which demonstrates that the RMSE value tended to decrease as the number of decision trees increased. Specifically, a stabilization trend of the RMSE over a value below 10.50 was observed starting at 67 decision trees. From this point onwards, the variation of RMSE was not remarkable, reaching an RMSE value equivalent to 10.37 at 100 decision trees. Therefore, the final number of decision trees was established at 100 in the present study, considering that this value is widely employed in different RF applications together with the fact that its application does not imply a high computational cost. With these values of mtry and ntrees, the model was trained using a 5-fold CV and the training set, obtaining RMSE and R^2^ of 10.37 and 0.9157 (Table 4). The model performance was established using the test set, achieving RMSE and R^2^ values of 9.782 and 0.9327 (Table 4), respectively. The performance of the RF model obtained was similar to that of PLSR in terms of the evaluation metrics used.

**Figure 7:**
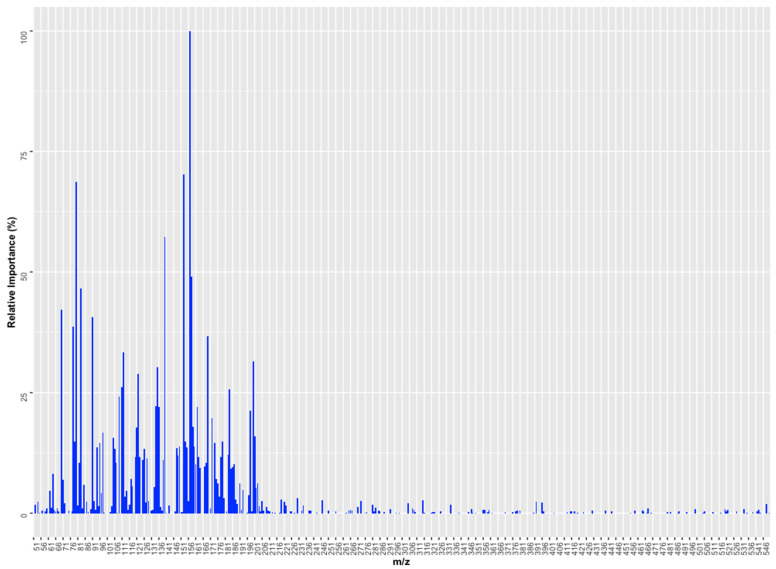
Graphical representation of the relative importance of *m*/*z* in the creation of the RF regression model.

Analogous to the PLS algorithm, the RF algorithm enables the identification of the most important variables in the construction of the model. To establish the relative importance of the variables, the *varImp* function of the caret package was used. A graphical representation of the relative importance of the predictors in the generated RF model is shown in Figure 7, which shows that *m*/*z* 157 presented a relative importance equivalent to 100%, followed by *m*/*z* 153 (70%) and 80 (69%). The selection of *m*/*z* 157 by the RF regression model agreed with the results obtained with the RF classifier, where this variable was also selected as important (relative importance higher than 70%). The variables selected by the RF regression model also differ from those obtained by the PLSR model. This discrepancy can be attributed to the fact that the significance of predictors is heavily dependent on the underlying model used. Different ML algorithms may interpret and weigh the importance of a predictor differently based on their internal mechanics and how they handle data. This means that what may be considered an important variable in one model (e.g., RF) might not be as crucial in another (e.g., PLSR), underscoring the idea that the relative importance of variables is model-specific. In other words, models trained with different ML algorithms may use a predictor differently.

##### Gaussian SVR

A model based on the SVR algorithm was created using the Gaussian kernel implementation. First, the hyperparameters C and σ were optimized. With exponentially growing sequences of C and σ, a grid search approach was used, taking values of log_2_C and log_2_σ from −10 to 10 every 0.5 units. Each combination of C and σ was performed using a 5-fold CV, and the criterion of lowest RMSE was used to choose the best choice. In addition, SVR has a third hyperparameter, ε, which regulates the learning rate and must be adjusted by the analyst. In this case, ε was maintained constant at 0.1. In Figure S7 (Supplementary Materials), which displays the contour plots for determining the optimal values of C and σ, it can be seen that the value of RMSE drops as log_2_C and log_2_σ increase. Finally, employing a total of 53 SVs, the optimal combination was determined to be a C value of 1024 (log_2_C = 10) and a value of 128 (log_2_σ = 7), resulting in an RMSE of 7.127 and an R^2^ of 0.9579 (Table 4). The model was validated using the test set after it had been tuned and trained, yielding RMSE and R^2^ values of 6.789 and 0.9667 (Table 4), respectively. Since lower RMSE and higher R^2^ values were obtained for both the 5-fold CV and test sets, the results obtained with the Gaussian SVR model are marginally better than those produced with the PLSR and RF regression models. This suggests that this model performs more accurately and efficiently when quantifying the proportion of odor in paraffin wax samples.

### 3.4. Web Application

From the obtained results, the Gaussian SVM and Gaussian SVR models presented a higher performance for discriminating and quantifying the odor grade in paraffin waxes. A Shiny App prototype (https://marta-barea.shinyapps.io/paraffin_odor_app/) has been developed to share these models and to show the advantages of the use of supervised ML techniques in the understanding of chemical information and automation of data processing. To correctly use this app, users are required to analyze their paraffin wax samples using the HS-GC/MS methodology described in this work. In turn, users must obtain the TIS for each sample and normalize the data to the base peak (maximum signal). Then, these data, in .csv/txt or .xlsx/xls format, will be uploaded to the app, where the models will be to discriminate the samples according to the odor intensity and quantify the percentage of odor. A test set has been introduced in the app to try it out. For this purpose, simply click on the “Download” button, where the .xlsx file with the test data can be downloaded. Once downloaded, the file can be uploaded in “File Input” by clicking on the “Browser” button. The app will indicate that the file is successfully uploaded, and the data will appear in the right region of the screen. Click on the “Submit” button for the models to perform the predictions. The results of the intensity and odor percentage estimation will be displayed afterward on the screen. This interactive web application has been created to be simple and intuitive to use. However, it can be improved and adapted to the needs and requirements of the users of the sector.

### 3.5. Discussions

The HS-GC/MS method, incorporating TIS and ML, presents significant advantages over ASTM D1833, electronic noses with gas sensors, and traditional GC/MS using TIC, particularly in the context of assessing odor levels in food-grade paraffin waxes. ASTM D1833, which employs a sensory panel for odor evaluation, can introduce subjectivity and variability due to its reliance on human sensory analysis. This traditional method is not only labor-intensive but also prone to inconsistencies across different testers and testing conditions. In stark contrast, HS-GC/MS with TIS and ML automates the process, significantly enhancing objectivity, repeatability, and accuracy, thus minimizing human intervention. Electronic noses equipped with gas sensors, although capable of detecting VOCs via sensor responses, can suffer from sensor drift and a limited range of detectable compounds. In comparison, HS-GC/MS with TIS and ML offers a more comprehensive aromatic profile, enabling fast and reliable analysis of the sample (Table 5). The ML component of this approach further facilitates the classification and quantification of complex odor profiles, a task that may prove challenging for electronic noses if the sensor array lacks the necessary diversity or specificity. Additionally, traditional GC/MS with TIC, while effective for analyzing known compounds, often fails to detect unknown volatiles that contribute to odors. TIS, on the other hand, captures a complete ion profile across the entire chromatographic run, providing a more exhaustive analysis. The integration of ML enhances this method’s ability to detect and quantify both known and unknown compounds, rendering HS-GC/MS with TIS and ML a versatile and powerful tool for comprehensive odor analysis.

As can be seen in Table 5, the integration of HS-GC/MS with TIS and advanced ML algorithms offers a compelling alternative for the automated, objective assessment of odor levels in food-grade paraffin waxes, providing significant improvements over traditional methods in terms of reliability, comprehensiveness, and efficiency.

## 4. Conclusions

The present research has successfully developed an automatic, effective, and objective methodology based on HS-GC/MS to characterize and quantify the odor grade in paraffin waxes. Specifically, both RSM–BBD and a univariate study have been applied for the optimization of the HS working conditions, obtaining accurate and repeatable results. The final method developed presents certain advantages based on the non-use of solvents and the analysis time, making it eco-friendly and applicable to routine analysis in petrochemical or agri-food laboratories using this material. On the other hand, the study of the relevant literature revealed that, to the best of our knowledge, this is the first time that TIS information has been employed as a strategy to characterize this parameter in paraffin wax. These chemical data, combined with the appropriate ML tools, allow for reproducible and fast processing of the results. Concretely, the unsupervised ML techniques, HCA and PCA, revealed the existence of a certain grouping tendency of the studied samples according to their odor level. Of the classification models generated, the one based on Gaussian SVM obtained excellent results (test set: 100% accuracy and kappa 1) for the discrimination of the odor level in the paraffin wax samples. On the other hand, of the regression algorithms explored for odor-grade quantification, the Gaussian SVR-based model obtained a higher performance (test set: 6.789 RMSE and 0.9667 R^2^). Furthermore, an interactive online application has been developed to share these two models with industry researchers and manufacturers as well as to make it simpler for this product to be controlled. These models can be retrained as more samples are examined. In this way, by creating a constantly updated database, the reality may be more precisely tuned to satisfy the quality control criteria of this PDP.

Future studies should focus on applying and evaluating this methodology across a broader range of real sample matrices to guarantee reliable prediction results in other samples. Moreover, expanding the database with real-time data from various sources will enhance the models’ predictive capabilities. 

## Figures and Tables

**Table 1:** Selected variables, their values, and the coded and uncoded levels used for the BBD.

Variable	−1	0	1
X_1_: Temperature (°C)	100	120	140
X_2_: Agitation (rpm)	250	500	750
X_3_: Sample quantity (g)	0.2	0.4	0.6

**Table 2:** Conditions of the BBD for the three variables, including experimental and predicted values.

Experiment	Factors			Response	
	X_1_	X_2_	X_3_	Euclidean Distance	
				Experimental	Predicted
1	−1	0	1	0.056794	0.0495423
2	1	−1	0	0.111667	0.103849
3	−1	−1	0	0.0546288	0.0542285
4	1	0	−1	0.0695383	0.07679
5	1	1	0	0.105074	0.105474
6	1	0	1	0.1244	0.124565
7	0	−1	−1	0.0580481	0.0586143
8	−1	1	0	0.0492514	0.0570693
9	0	−1	1	0.0918902	0.0995423
10	0	1	1	0.0831786	0.0826123
11	−1	0	−1	0.0539537	0.0537878
12	0	1	−1	0.0876625	0.0800104
13	0	0	0	0.0808236	0.0831342
14	0	0	0	0.0825085	0.0831342
15	0	0	0	0.0898495	0.0831342
16	0	0	0	0.0641407	0.0831342
17	0	0	0	0.0892545	0.0831342
18	0	0	0	0.0922287	0.0831342

**Table 3:** Analysis of variance (ANOVA) of the quadratic model adjusted to the discrimination of odor grade in paraffin waxes, with degrees of freedom set at 1 across all experiments.

Variable	Factor	Sum of Squares	Mean Square	*F*-Value	*p*-Value
Temperature	X_1_	0.00480448	0.00480448	45.18	0.0011
Agitation	X_2_	0.00000997329	0.00000997329	0.09	0.7718
Sample quantity	X3	0.000947424	0.000947424	8.91	0.0306
Temperature: Temperature	X_1_X_1_	0.0000534923	0.0000534923	0.50	0.5099
Temperature: Agitation	X_1_X_2_	3.69418E−7	3.69418E−7	0.00	0.9553
Temperature: Sample quantity	X_1_X_3_	0.000676543	0.000676543	6.36	0.0530
Agitation: Agitation	X_2_X_2_	0.00000118999	0.00000118999	0.01	0.9199
Agitation: Sample quantity	X_2_X_3_	0.000367222	0.000367222	3.45	0.1223
Sample quantity: Sample quantity	X_3_X_3_	0.0000522889	0.0000522889	0.49	0.5145

**Table 4:** Results obtained for each regression model in the discrimination of odor grade in paraffin waxes.

		5-Fold CV		Test Set	
Model	Hyperparameters	RMSE	R^2^	RMSE	R^2^
PLSR	No. components = 7	9.797	0.9330	9.227	0.9328
RF	mtry = 167; No. trees = 100	10.37	0.9157	9.782	0.9327
RBF-SVR	C = 1024; γ = 128; ε = 0.1	7.127	0.9579	6.789	0.9667

**Table 5:** HS-GC/MS with TIS and ML vs. other methods.

Method	Merits	Disadvantages	Applications	Analysis Time	Solvents Used
HS-GC/MS with TIS and ML	High accuracy, comprehensive analysis, automated processing	Setup for ML algorithms and training data required	Quality control in industries like food-grade materials	Short to medium	None (direct analysis)
ASTM D1833	Standardized, simple implementation	Subjective, requires human panel, potential for inconsistency	Quality assessment in petroleum product industries	Short to medium	None (sensory analysis)
Electronic Noses with Gas Sensors	Quick, real-time monitoring, portable	Sensor stability issues, limited compound detection range	Broad applications from food quality to environmental monitoring	Very short	None (sensor-based)
GC/MS with TIC	Accurate for known compounds, reproducible	Limited detection of unknowns, complex setup and calibration	Detailed VOC analysis in chemical and environmental sciences	Medium to long	Varies with sample preparation

## Data Availability

The original contributions presented in the study are included in the article/Supplementary Materials, further inquiries can be directed to the corresponding author.

## Supplementary Materials

The following supporting information can be downloaded at https://www.mdpi.com/article/10.3390/foods13091352/s1: Figure S1: Experimental versus observed values obtained with the Box–Behnken Design; Figure S2: 2D contour plot showing the evolution of the 5-fold CV accuracy in the search for the optimal values of C and σ for the Gaussian SVM classifier; Figure S3: Evolution of 5-fold CV accuracy as a function of the number of decision trees; Figure S4: Graphical display of the 20 most important *m*/*z* and their relative importance in the RF classifier; Figure S5: Evolution of RMSE as a function of the number of components using 5-fold CV; Figure S6: Evolution of 5-fold CV RMSE as a function of the number of decision trees; Figure S7: 2D contour plot showing the evolution of the 5-fold CV RMSE in the search for the optimal values of C and σ for the Gaussian SVR model.

## Abbreviations

HS-GC/MS	Headspace Gas Chromatography with Mass Spectrometry
ML	Machine Learning
HS	Headspace
GC	Gas Chromatography
MS	Mass Spectrometry
HCA	Hierarchical Cluster Analysis
PCA	Principal Component Analysis
SVM	Support Vector Machine
SVR	Support Vector Regression
RF	Random Forest
PLSR	Partial Least Squares Regression
BBD	Box–Behnken Design
RSM	Response Surface Methodology
VOCs	Volatile Organic Compounds
SVOCs	Semi-Volatile Organic Compounds
TIS	Total Ion Spectra
TIC	Total Ion Chromatogram
SPME	Solid-Phase Microextraction
TD	Thermal Desorption
ASTM	American Society for Testing and Materials
FDA	Food and Drug Administration
ANOVA	Analysis of Variance
CV	Coefficient of Variation
ELM	Extreme Learning Machine
*m*/*z*	Mass-to-Charge Ratio
