# Supplementary figures and images for: Development of a Novel HS-GC/MS Method Using the Total Ion Spectra Combined with Machine Learning for the Intelligent and Automatic Evaluation of Food-Grade Paraffin Wax Odor Level
Source: Foods. 2024 Apr 27;13(9):1352. doi: 10.3390/foods13091352 (PMC11083247; doi:10.3390/foods13091352)

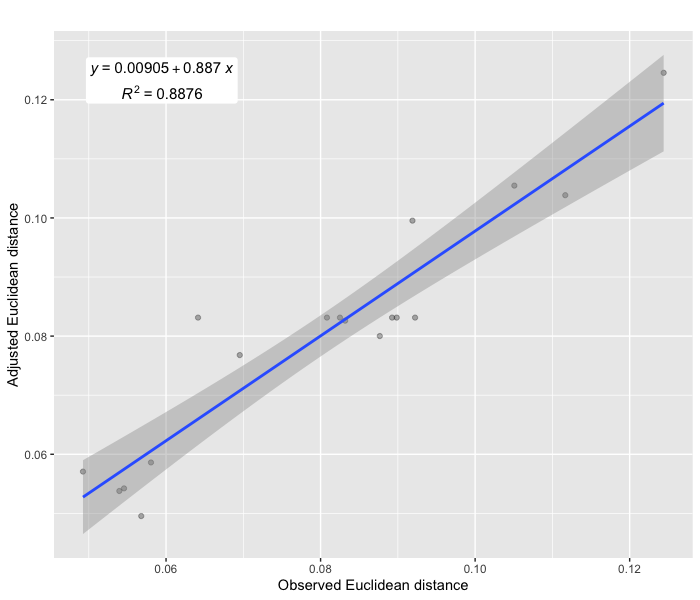

Supplement: Supplementary file 1 [file foods-13-01352-s001.zip › Fig.1S.tiff]

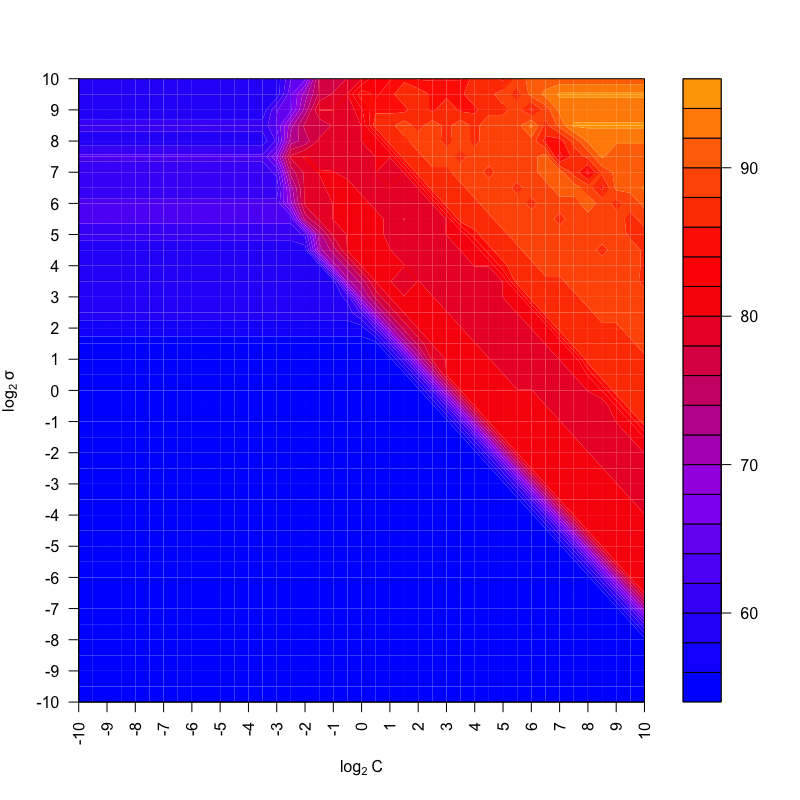

Supplement: Supplementary file 1 [file foods-13-01352-s001.zip › Fig.2S.tiff]

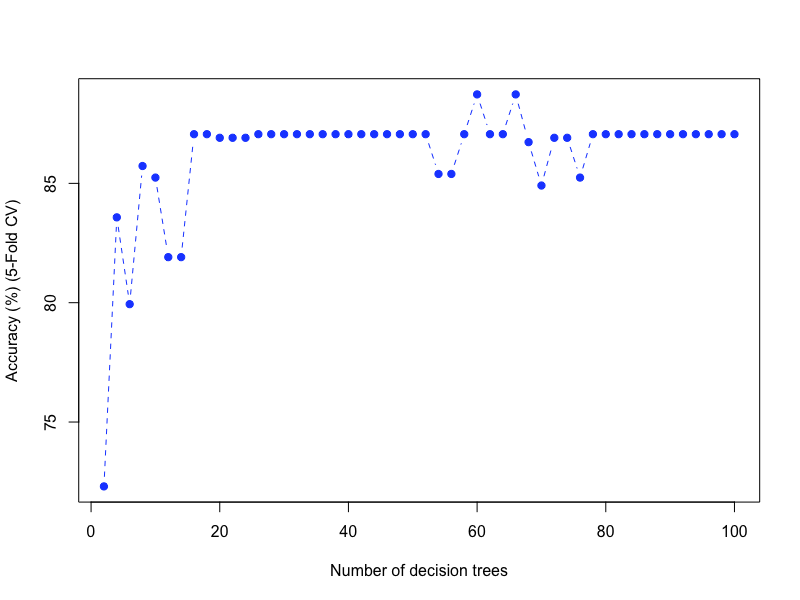

Supplement: Supplementary file 1 [file foods-13-01352-s001.zip › Fig.3S.tiff]

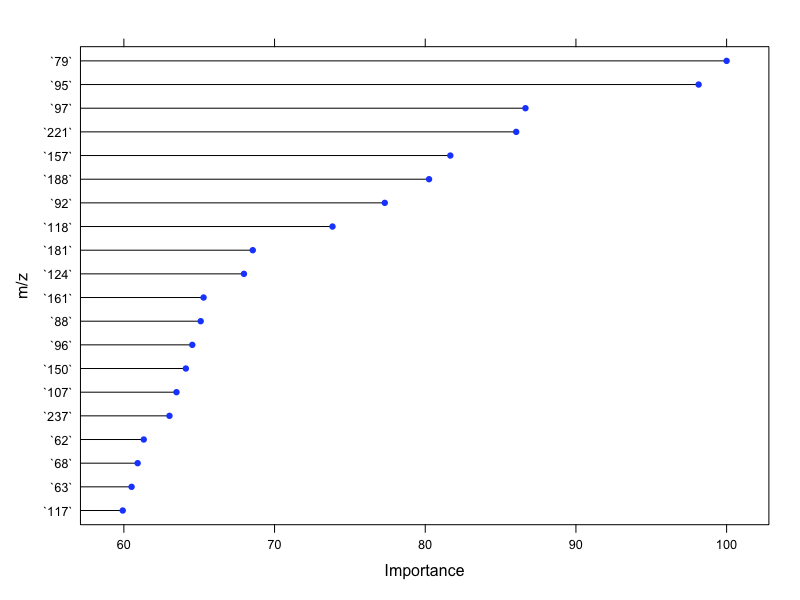

Supplement: Supplementary file 1 [file foods-13-01352-s001.zip › Fig.4S.tiff]

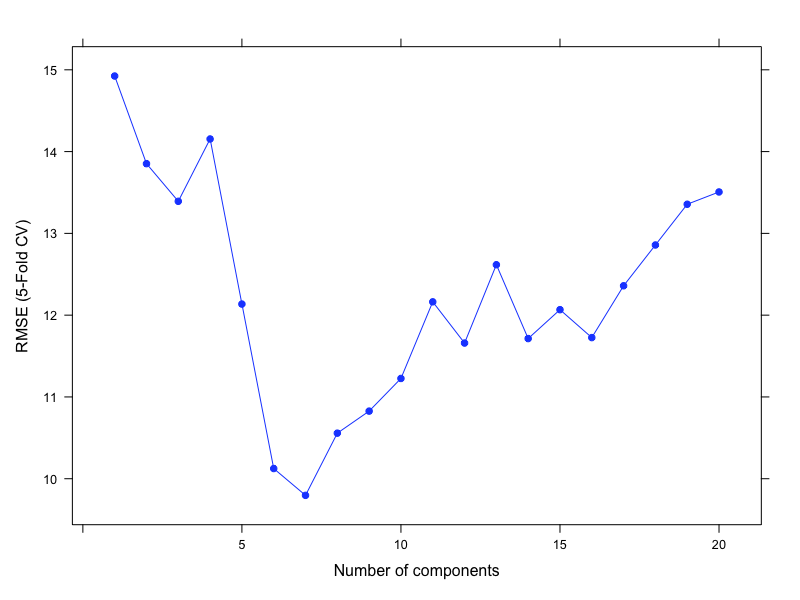

Supplement: Supplementary file 1 [file foods-13-01352-s001.zip › Fig.5S.tiff]

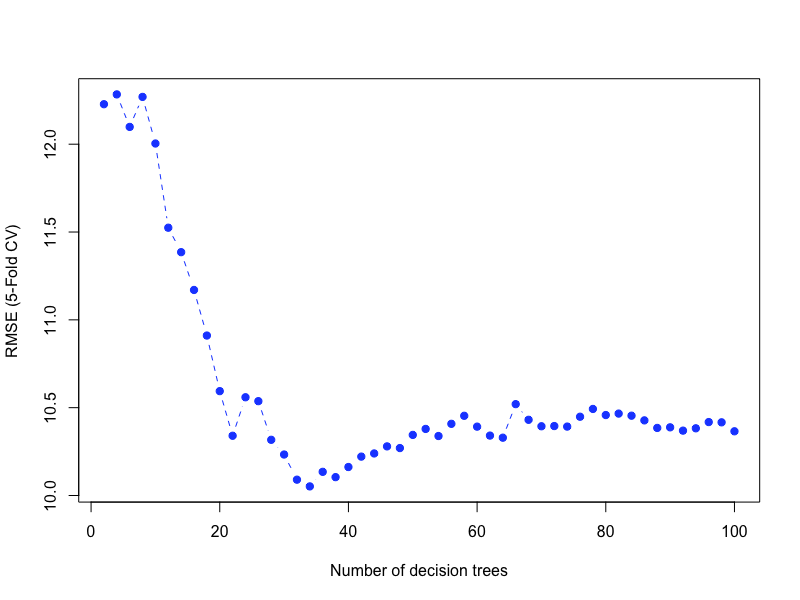

Supplement: Supplementary file 1 [file foods-13-01352-s001.zip › Fig.6S.tiff]

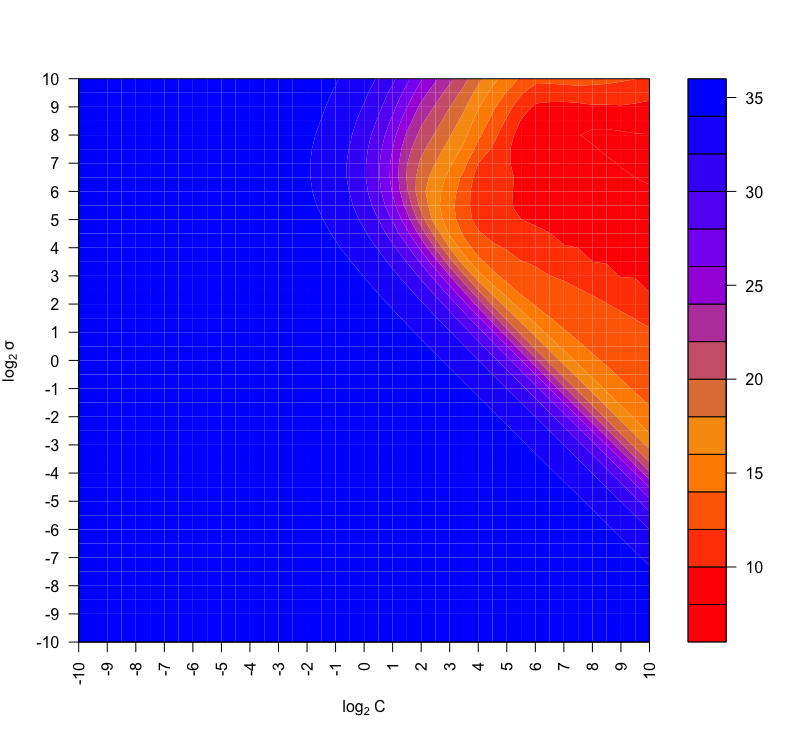

Supplement: Supplementary file 1 [file foods-13-01352-s001.zip › Fig.7S.tiff]
